# Deletion of exon 8 from the EXT1 gene causes multiple osteochondromas (MO) in a family with three affected members

**DOI:** 10.1186/s40064-016-1695-6

**Published:** 2016-01-22

**Authors:** Lei Zhuang, Simon D. Gerber, Stefan Kuchen, Peter M. Villiger, Beat Trueb

**Affiliations:** Department of Clinical Research, University of Bern, Murtenstrasse 35, 3008 Bern, Switzerland; Institut IMAGINE, 75015 Paris, France; Department of Rheumatology, University Hospital, 3010 Bern, Switzerland

**Keywords:** Multiple osteochondromas (MO), Hereditary multiple exostoses (HME), Exostosin-1 (EXT1), Glycosyltransferase, Genomic deletion

## Abstract

Multiple osteochondromas (also called hereditary multiple exostoses) is an autosomal dominant disorder characterized by multiple cartilaginous tumors, which are caused by mutations in the genes for exostosin-1 (EXT1) and exostosin-2 (EXT2). The goal of this study was to elucidate the genetic alterations in a family with three affected members. Isolation of RNA from the patients’ blood followed by reverse transcription and PCR amplification of selected fragments showed that the three patients lack a specific region of 90 bp from their EXT1 mRNA. This region corresponds to the sequence of exon 8 from the EXT1 gene. No splice site mutation was found around exon 8. However, long-range PCR amplification of the region from intron 7 to intron 8 indicated that the three patients contain a deletion of 4318 bp, which includes exon 8 and part of the flanking introns. There is evidence that the deletion was caused by non-homologous end joining because the breakpoints are not located within a repetitive element, but contain multiple copies of the deletion hotspot sequence TGRRKM. Exon 8 encodes part of the active site of the EXT1 enzyme, including the DXD signature of all UDP-sugar glycosyltransferases. It is conceivable that the mutant protein exerts a dominant negative effect on the activity of the EXT glycosyltransferase since it might interact with normal copies of the enzyme to form an inactive hetero-oligomeric complex. We suggest that sequencing of RNA might be superior to exome sequencing to detect short deletions of a single exon.

**Electronic supplementary material:**

The online version of this article (doi:10.1186/s40064-016-1695-6) contains supplementary material, which is available to authorized users.

## Background

Multiple osteochondromas (MO), also called hereditary multiple exostoses (HME), is a genetic, autosomal dominant disorder characterized by multiple benign cartilaginous tumors. These tumors (or exostoses) form next to the growth plate of long bones, ribs and pelvis and protrude into the adjacent perichondrium and neighboring tissue (Huegel et al. 2013). Patients with MO are frequently of short stature and show shortened forearms, changes in the angle of fingers and ankle and unequal length of limbs. Most of the patients have to undergo repetitive surgery to remove some of the exostoses because they cause pain or functional impairment (Pedrini et al. 2011). The most serious complication of MO is a malignant transformation into a secondary chondrosarcoma, which occurs in 1–5 % of the patients.

MO is caused by heterozygous inactivating mutations of the genes for exostosin-1 (EXT1) or exostosin-2 (EXT2). The majority of these mutations are nonsense, frame shift and splice-site mutations, but there are also missense mutations and deletions (Jennes et al. 2009; Pedrini et al. 2011). About 65 % of the MO mutations occur in EXT1 and 25 % in EXT2. The residual 10 % of the patients do not display any overt mutations in EXT1 or EXT2. Recent results suggest, however, that some of these cases might be explained by somatic, mosaic mutations in the EXT genes (loss of heterozygosity) and/or by mutations in some of the large introns (Szuhai et al. 2011; Waaijer et al. 2013).

EXT1 and EXT2 code for glycosyltransferases in the Golgi apparatus that are involved in the formation of heparan sulfate chains for proteoglycans (Esko and Selleck 2002; Nadanaka and Kitagawa 2008). Both enzymes are typical type II transmembrane proteins with the C-terminal part of the protein outside of the membrane. The growth plate of long bones is known to contain large amounts of heparan sulfate proteoglycans, mainly syndecan, glypican and perlecan (Farach-Carson et al. 2005). EXT1 and EXT2 belong to a small family of related proteins, which also includes the three exostosin-like proteins EXTL1, EXTL2 and EXTL3. With the exception of EXTL2, all EXT proteins contain a transmembrane domain at the N-terminal end, an Exostosin interaction domain in the center and a catalytic domain at the C-terminal end (Additional file 1: Fig. S1). EXTL2 in contrast contains only the transmembrane domain and the catalytic domain. EXT1 and EXT2 form a hetero-oligomeric complex (Kobayashi et al. 2000; McCormick et al. 2000; Senay et al. 2000) that adds repeating units of glucuronic acid (GlcA) and N-acetyl-glucosamine (GlcNAc) to the nascent chain of heparan sulfate. EXTL1, EXTL2 and EXTL3 on the other hand, appear to be involved in the addition of the first carbohydrate residue to the linker tetrasachararide of the proteoglycan core protein (Nadanaka and Kitagawa 2008).

Loss-of-function mutations in one allele of the EXT genes cause a significant decrease of glycosyltransferase activity. Why the loss of a single copy of EXT1 or EXT2 leads to a nearly complete loss of enzymatic activity is not yet clear (Hall et al. 2002). It is possible that the mutated proteins interact with normal proteins to form an inactive hetero-oligomeric complex. Furthermore, studies with isolated chondrocytes from patients showed that cells with an EXT mutation have a significantly decreased survival rate (Hecht et al. 2005). At any rate, the decrease in glycosyltransferase activity leads to a general deficiency of heparan sulfate in the growth plate. This deficiency causes an increase in the distribution range of signaling proteins, including Ihh, BMP, FGF and Wnt. Diffusion of these signaling proteins is usually restricted by binding to heparan sulfate proteoglycans found on cell surfaces and in the extracellular matrix. Finally, the increased distribution range of the growth factors might initiate ectopic chondrogenesis and exostosis formation.

In the present work we have studied a patient with MO from a family containing three affected members. The patient revealed a localized deletion of a single exon of EXT1. Deletions, especially deletions encompassing only a single exon have rarely been found with MO patients, probably because such deletions are more difficult to detect by conventional methods.

## Results

### Description of patients

**Fig. 1:**
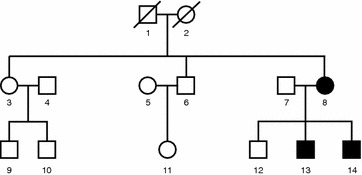
Pedigree of the family of patient #13. Exostoses typical of MO were found only with mother #8 and her sons #13 and #14. Son #12, father #7, grandparents #1 and #2, aunt #3 and uncle #6 did not show any clinical signs of the disease

**Fig. 2:**
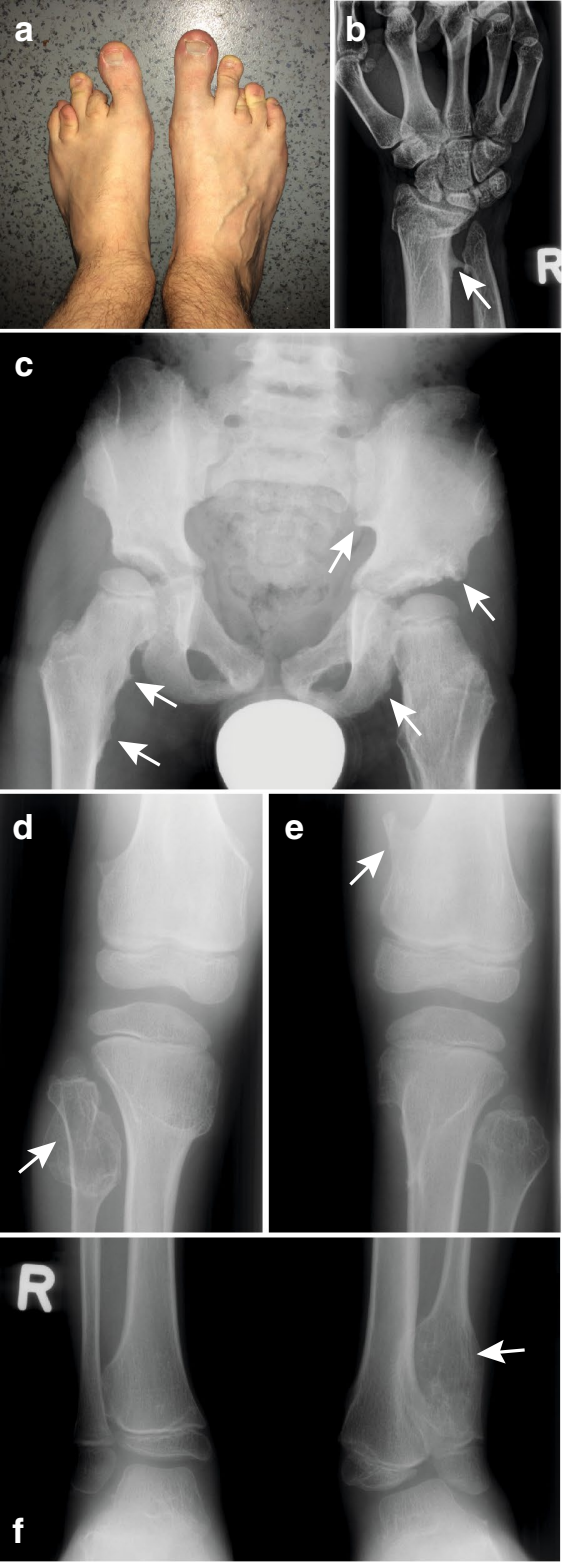
Phenotype of patient #13. A picture of the feet (**a**; age 34) shows unequal length of toes. An X-ray image of the right hand (**b**; age 19) shows an exostosis of the distal radius (*arrow*) and a shortened ulna. The X-ray of the pelvis (**c**; age 6) exhibits multiple exostoses on the pelvis and on the proximal femurs, which cause a locally irregular, tubercular aspect of the bone surface (*arrows*; compare the indicated regions with the contralateral side). The X-ray of the right knee (**d**; age 6) shows an osteochondroma on the head of the fibula (*arrow*). The X-ray of the left knee (**e**; age 6) demonstrates a prominent exostosis on the medial part of the femur (*arrow*). The X-ray of the lower legs (**f**; age 7) shows an osteochondroma at the distal end of the left fibula

The pedigree of the family of the index patient #13 is shown in Fig. 1. The index patient, who is currently 34 years old, exhibited a length difference of his toes already at birth (Fig. 2a). At the age of 12, he was diagnosed to suffer from MO based on progressive exostoses at numerous locations, including toes, ankles, knees, hips, ribs, fingers, wrist, elbows and shoulders (Fig. 2b–f). Repeated surgical interventions were required to correct functional impairment.

The currently 29-year-old younger brother (#14) of the index patient was diagnosed at the age of 12. Like his older brother, he had multiple exostoses, some of which required surgical resection. He shows shortened legs and forearms and has a mild walking impairment.

The 61-year-old mother (#8) of the index patient was diagnosed at age 10. Most of her limbs are shorter than normal. She underwent multiple surgical interventions before the age of 18 in order to remove exostoses. Due to a length difference of her legs she has severe walking impairment.

Based on these clinical findings it is likely that mother #8 harbors a de novo mutation, which was passed on to her sons #13 and #14. This mutation might have occurred early during development or alternatively, it was already present in the germline of one of the grandparents #1 or #2.

### Identification of the mutation

**Fig. 3:**
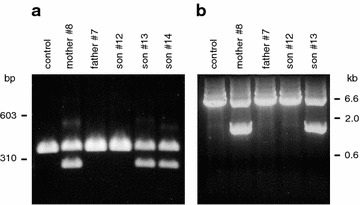
Localization of the mutation. Agarose gels stained with ethidium bromide are shown. **a** PCR amplification of the region corresponding to nucleotides 2370-2723 of the exostosin-1 mRNA (NM_000127.2) yielded two fragments of 354 and 264 bp, respectively, with samples from the mother #8 and her sons #13 and #14, but a single fragment with samples from an unrelated control as well as from father #7 and the healthy brother #12. **b** Long-range PCR of a fragment corresponding to the region from intron 7 to intron 8 of the EXT1 gene yielded two fragments of 5.6 and 1.4 kb, respectively, with samples from mother #8 and son #13, but a single fragment with samples from father #7 and the healthy son #12

**Fig. 4:**
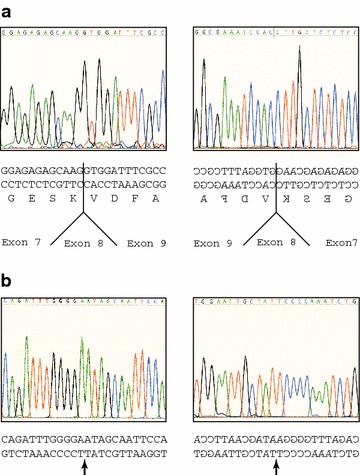
Sequencing of mutated exostosin-1 from patient #13. **a** Parts of the original sequencing chromatograms of the EXT1 cDNA are shown together with the DNA sequence of both strands and the deduced amino acid sequence in single letter code. The *left panel* shows sequencing from the 5′ end, the *right panel* sequencing from the 3′ end (complementary strand). Note that the cDNA sequence of patient #13 leads from the region of exon 7 directly into the region of exon 9. **b** Parts of the sequencing chromatograms of the genomic EXT1 DNA are depicted together with the DNA sequence. The position where a deletion is found when compared to the EXT1 gene (NC_000008.11) is indicated by an *arrow*. The *left panel* shows sequencing from the 5′ end of the EXT1 gene, the *right panel* sequencing from the 3′ end

Since the majority of the cases of MO can be explained by heterozygous mutations in EXT1 or EXT2 (Huegel et al. 2013; Jennes et al. 2009), we set out to determine the sequences of the open reading frames of these two genes from the index patient #13. Total RNA was extracted from freshly drawn blood and transcribed into cDNA. PCR amplification of overlapping fragments from these cDNAs (see Additional file 1: Table S1) followed by DNA sequencing established the complete sequences of the EXT1 and EXT2 mRNAs. No mutation or polymorphism was found in EXT2. However, two relatively common polymorphisms were identified in EXT1, namely SNP rs11546829 A/G at position 1838 of the mRNA (global minor allele frequency MAF 0.173) and SNP rs7837891 C/T at position 2534 (MAF 0.338). Neither of these polymorphisms will lead to any change in the amino acid sequence. In addition, a primer pair for region 2370-2723 of EXT1 yielded two products that ran as two clearly separated bands on an agarose gel, a 354 bp fragment migrating with the band from a control cDNA (wildtype allele) and a novel 264 bp fragment (Fig. 3a). The two fragments were roughly obtained in a 1:1 ratio, suggesting that the 264 bp fragment corresponded to the mutated allele. As expected, the novel 264 bp fragment was also obtained with RNA from the mother #8 and the younger brother #14, but not with RNA from the father #7 or the healthy brother #12. Finally, sequencing of the 264 bp fragment showed that the patients were lacking nucleotides 2406-2495 of the EXT1 mRNA (NM_000127.2). The absence of these 90 bp could be verified on the coding as well as noncoding strand (Fig. 4a).

**Fig. 5:**
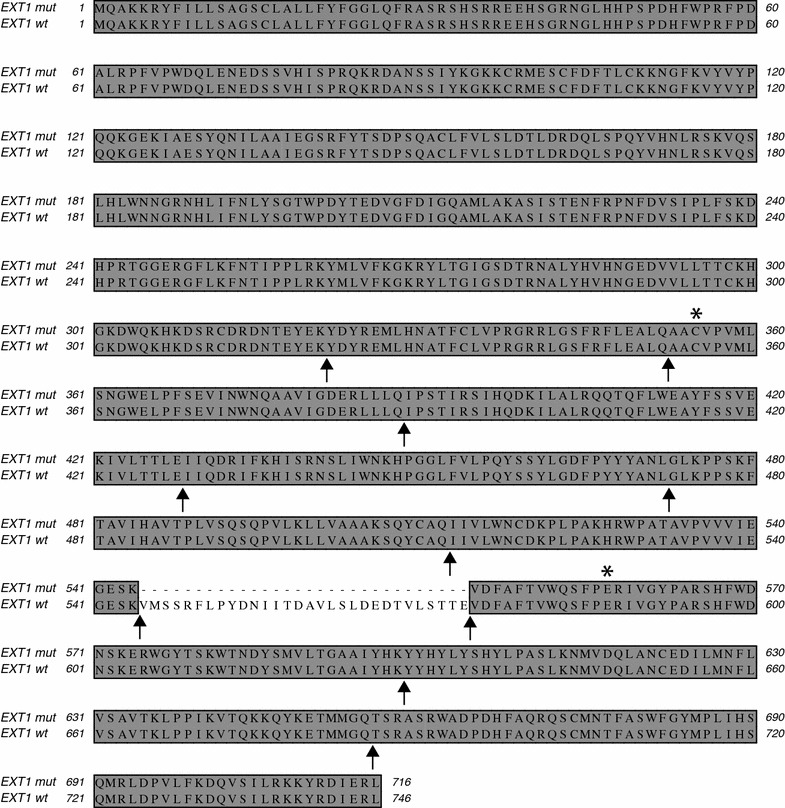
Sequence of mutant exostosin-1 from patient #13. The amino acid sequence derived from the mRNA sequence was compared with the published protein sequence of exostosin-1 (NP_000118). The relative locations of introns in the EXT1 gene are indicated by *arrows*. Note that the sequence of patient #13 exactly lacks the region of the protein, which is derived from exon 8. The positions of two polymorphisms (rs7837891 and rs11546829) that were found in the cDNA are marked by *asterisks*. They did not lead to any change in the amino acid sequence

Nucleotides 2406-2495 of the mRNA correspond exactly to the region of the protein that is encoded by exon 8 of the EXT1 gene (Fig. 5). We therefore analyzed the splice sites of exon 8 using genomic DNA purified from the blood of the index patient. However, neither the donor splice site nor the acceptor splice site showed any alteration when compared to the published EXT1 gene sequence (NC_000008.11). It was therefore likely that our patient suffered from a localized deletion of exon 8.

**Fig. 6:**
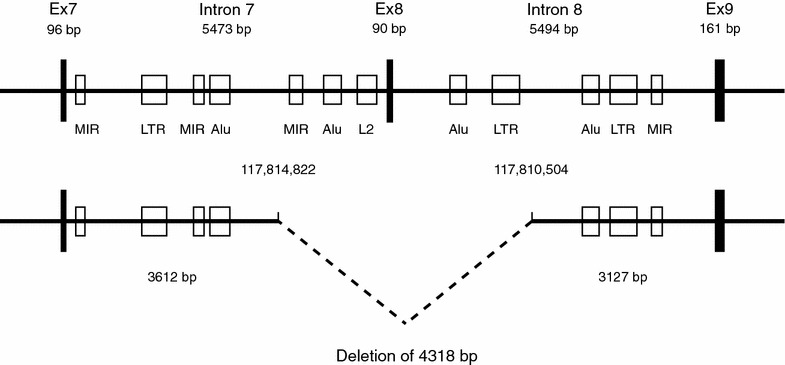
Schematic drawing of the mutated region of the EXT1 gene at chromosomal location 8p24 from patient #13. Exons (Ex7, Ex8, Ex9) are given as *black boxes*, introns as *straight lines*. Repetitive elements (MIR, LTR, Alu, L2) are depicted as *open boxes*. The wildtype allele is shown at the *top*, the mutant allele at the *bottom*. Note that patient #13 contains a deletion of 4318 bp that includes exon 8. The two deletion breakpoints occur close to, but not within repetitive elements

To confirm this notion, we performed long-range PCR with genomic DNA. Utilizing two primers that annealed to intronic sequences adjacent to exon 7 and exon 9 (Additional file 1: Table S1), we were able to amplify a fragment of 8 kb from an unrelated control and from father #7. However, with DNA from the index patient #13 we obtained two fragments, one of 8 kb (wildtype allele) and another one of 3.7 kb, which might include the deletion (not shown). This observation was confirmed with a different primer set, which annealed closer to the center of the two introns. These primer pair amplified a fragment of 5.6 kb from the unrelated control, from father #7 and from the healthy brother #12, but two fragments of 5.6 kb (wildtype allele) and 1.3 kb from the index patient #13 (Fig. 3b). DNA sequencing of the entire 1.3 kb fragment on both strands (Fig. 4b) confirmed a deletion of 4318 bp. This deletion was situated between position 117,814,822 and 117,810,504 of the EXT1 gene and encompassed exon 8 and part of the flanking introns. The region contained several repetitive elements, such as MIR, Alu, L2 and LTR (Fig. 6). The same deletion was also found with genomic DNA from the mother #8 and the younger brother #14 (not shown).

### Mechanism of deletion

**Fig. 7:**
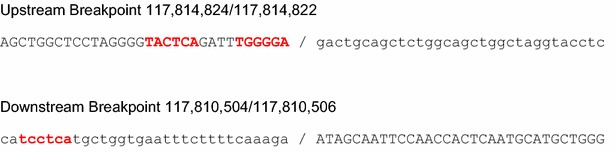
Deletion breakpoints of the mutant allele from patient #13. The deleted sequence is shown in *lower case letters*. The upstream breakpoint (relative to the orientation of the EXT1 gene) occurs at position 117,814,822 of the EXT1 gene, the downstream breakpoint at position 117,810,504. Since the deletion breakpoints contain a microrepeat of 2 nucleotides (GA), the breakpoints are slightly ambiguous. Note that the EXT1 gene is located on the lower strand of chromosome 8 at region 8p24. Close to the breakpoints, three copies of the deletion hotspot consensus sequence TGRRKM are found (highlighted in *red*)

At least five mechanisms have been suggested to explain the occurrence of a genomic deletion, including (1) homologous recombination (HR), (2) non-homologous end-joining (NHEJ), (3) microhomology-mediated replication-dependent recombination (MMRDR), (4) long interspersed L1-mediated retrotransposition and (5) telomere healing (Chen et al. 2010). Since in our case no sequence homology was observed at the two deletion breakpoints and since these breakpoints did not occur within an L1 element, three of the above mentioned possibilities could be ruled out. Furthermore, the EXT1 gene is not located at a telomere end but in chromosomal region 8p24. On the other hand, the upstream deletion breakpoint at position 117,814,822 contained the sequence TGGGGA (Fig. 7), which matches the general sequence TGRRKM of the deletion hotspot consensus sequence described in the literature (Krawczak and Cooper 1991; Abeysinghe et al. 2003). Another 10 bp upstream, the same hotspot sequence occurred on the lower DNA strand. Furthermore, a third copy of the hotspot sequence was found at the downstream breakpoint on the lower strand. Thus, our case is most consistent with the classical mechanism of NHEJ. It is conceivable that DNA polymerase paused at the deletion breakpoint hotspot sequences during replication (Krawczak and Cooper 1991). This replication arrest might have prolonged the single-stranded state of the DNA and made it vulnerable to illegitimate recombination. Finally, the mutated allele was inherited from mother #8 to her sons #13 and #14.

## Discussion

In this study, we have investigated the molecular cause of MO syndrome in a family with three affected members. By PCR amplification and DNA sequencing, we were able to identify a discrete deletion of 4318 bp that includes exon 8 of the EXT1 gene and parts of the flanking introns.

In contrast to many other studies, which rely on exon sequencing of genomic DNA, we have started from purified RNA of the index patient and analyzed the sequences of the EXT1 and EXT2 mRNAs. We reasoned that leukocytes from the patient’s blood must contain heparan sulfate proteoglycans on their cell surfaces and consequently contain detectable levels of mRNA for these glycosyltransferases. It is likely that we would have missed the relatively short deletion of exon 8 if we had used exome sequencing. In fact, a primer pair flanking this exon amplified a fragment with the expected size from the intact allele, whereas no product was obtained from the mutant allele.

Large genomic deletions spanning several kb of genomic DNA are usually detected by fluorescent in situ hybridization (FISH). However, short deletions spanning only a few hundred bp are difficult to identify by this method due to its limited sensitivity. In this case, quantitative comparative hybridization to a gene array containing genomic DNA fragments (array-CGH) is the method of choice. But again, short deletions as in our case are difficult to detect by gene arrays, especially when these arrays are based on common SNPs with poor resolution. In order to detect short deletions, some researchers have developed special arrays with “tiling resolution” that contained synthetic DNA fragments based on the genomic sequence of interest (Szuhai et al. 2011). However, such arrays are not commercially available and have to be prepared individually for each project.

The most common mutations identified in MO patients are frameshift mutations (44 %), nonsense mutations (24 %), and splice site mutations (11 %). In most cases, these mutations result in premature termination of translation and polypeptide truncation (Jennes et al. 2009). Furthermore, it is likely that the affected mRNAs are degraded by nonsense-mediated decay NMD (Miller and Pearce 2014). Deletions, especially deletions of a single exon as in our case, are rather rare. Jennes et al. (2008) analyzed a cohort of 63 MO patients and found only 3 patients with EXT1 deletions. Furthermore, we are aware of only one additional family with a deletion of exon 8 alone, which has been published in the literature (Jennes et al. 2008, 2011). It is of interest to note that the exact deletion breakpoints differ between our family (117,814,822-117,810,504) and the one reported in the literature (117,815,648-117,809,763).

Exon 8 is 90 bp long and codes for 30 amino acids. Since it is flanked by splice sites of phase 0, deletion will not change the reading frame. Thus, the transcribed mRNA will contain the normal stop codon and the normal 3′UTR; consequently it will not be recognized by NMD for degradation. In fact, we observed roughly equimolar amounts of the cDNAs from the wildtype allele and from the mutated allele. It is possible that this fact makes the mutation particularly devastating as discussed in the following.

**Fig. 8:**
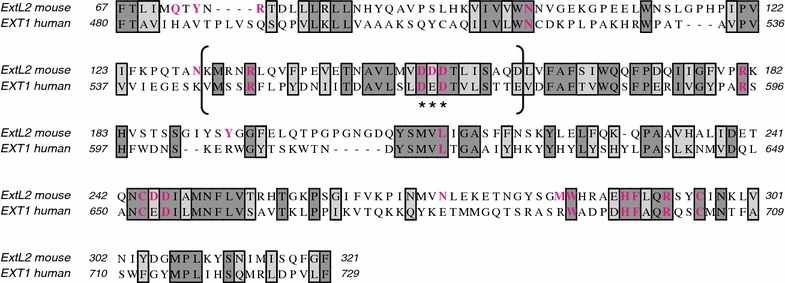
Alignment of the amino acid sequences of mouse ExtL2 (NP_001156986) and human EXT1 (NP_000118). Homologies are* boxed* and* shaded* according to identity (*dark grey*) and similarity (*light grey*). Although the two EXT proteins do not show much sequence homology, most of the critical amino acid residues, which are known to interact with the catalytic manganese ion and the UDP-sugar, are conserved (marked in *red*). The sequence encoded by exon 8 of the human EXT1 gene is given in *brackets*. It harbors the DXD signature (indicated by three *asterisks*) present in all UDP-dependent glycosyltransferases as well as a critical arginine residue that forms a hydrogen bond with the donor sugar

The 3D structures of EXT1 and EXT2 are not yet known. However, the 3D structure of the homologous glycosyltransferase ExtL2 from mouse has been elucidated by X-ray crystallography (Pedersen et al. 2003). Although the primary sequences of the individual members of the EXT family show rather a low degree of conservation, most of the residues involved in binding of the donor sugar (in this case UDP-GlcNAc) and the catalytic metal ion (Mn^2+^) are highly conserved (Fig. 8). It has therefore been suggested that the pocket for binding of the donor UDP-sugar should exhibit a similar structure in all members of the EXT family (Pedersen et al. 2003).

In a pairwise alignment (Fig. 8) the portion of the human EXT1 sequence, which is encoded by exon 8 but missing in our patients, aligns with residues 131-160 of mouse ExtL2. This is exactly the region that would interact with the UDP-sugar and the essential manganese ion. The DXD motif, which represents the signature of all UDP-sugar dependent glycosyltransferases (Breton and Imberty 1999), is found in mouse ExtL2 at residues 151-153. The two aspartate residues have been shown to interact with Mn^2+^ and the UDP-sugar and to position them in the proper orientation for catalysis (Pedersen et al. 2003). This motif is conserved as 565-DED-567 in human EXT1. Furthermore, residue R135 of mouse ExtL2, which forms a hydrogen bond with the donor sugar, is conserved as R549 in human EXT1 (Fig. 8). Based on this alignment, it is obvious that the EXT1 protein of our patients must be enzymatically inactive.

Quantitative analysis of the glycosyltransferase activity of mutated EXT1 proteins from several MO patients has shown that the residual enzyme activity was much lower than 50 % as would be expected in the heterozygous state (Hecht et al. 2005; Cheung et al. 2001). Theoretically there are two ways to explain why mutation of a single EXT1 copy might result in MO in a dominant fashion. Either, the residual glycosyltransferase activity is not sufficient to fulfill the function of the enzyme (haploinsufficiency), or alternatively, the mutated protein exerts a dominant negative effect on the activity of the wildtype protein. Work with knockout mice has provided evidence against the first possibility. Homozygous Ext1 knockout mice (expected Ext enzyme levels 0 %) are lethal and die at embryonic day E8.5, indicating the importance of heparan sulfate for embryonic development (Lin et al. 2000). However, heterozygous knockout mice (expected Ext enzyme levels 50 %) are largely normal and lack an obvious skeletal phenotype, thus ruling out haploinsufficiency in mice (Hilton et al. 2005). Likewise, homozygous Ext2 knockout mice are embryonically lethal. However, heterozygous Ext2 knockout mice develop, although with very low frequency, exostosis-like protrusions along the ribs, suggesting that a partial loss of the Ext glycosyltransferase activity may be sufficient for exostosis formation (Stickens et al. 2005). Moreover, compound heterozygous mice lacking one allele of Ext1 plus one allele of Ext2 frequently exhibit exostoses on the long bones (Zak et al. 2011). These mice should possess approximately 25 % of the Ext enzyme levels when compared to wildtype littermates. Thus, a significant, but not necessarily a complete loss of Ext protein expression is sufficient to explain exostoses formation.

Our MO patients contain a heterozygous deletion in EXT1 and show only a relatively small reduction of the normal EXT1 mRNA levels (Fig. 3a). Mutation of the second allele (loss of heterozygosity) in somatic cells would explain a further decrease of EXT enzyme activity, but loss of heterozygosity is very rare and has been ruled out in the majority of the MO patients (Hall et al. 2002). It is therefore likely that the mutant protein encoded by the affected allele exerts a dominant negative effect on the intact protein encoded by the wildtype allele.

How can we explain a dominant negative effect of the mutated EXT1 protein? EXT1 is known to form an oligomeric complex with EXT2 (Kobayashi et al. 2000; McCormick et al. 2000; Senay et al. 2000). It is likely that this complex contains several copies of EXT1 and EXT2 (e.g. stoichiometry 2:2 or 4:4) because it has to interact with the nascent chain of heparan sulfate that consists of at least four sugar residues, the starting tetrasaccharide (Nadanaka and Kitagawa 2008). It is therefore conceivable that incorporation of a mutated copy of EXT1 into such an assembly will render the entire complex inactive. One false copy could therefore compromise the activity of several normal copies of the polypeptide. Since the N-terminal domain of EXT1, which is involved in protein–protein interactions, remains intact in our case, it is likely that the mutated enzyme will still be incorporated into the final complex. Faithful incorporation of a mutant protein into an oligomeric complex would thus explain a dominant negative effect. However, it should be emphasized that there might be additional ways to explain a dominant negative effect.

## Conclusion

We have demonstrated that sequencing of the mRNA, rather than the genomic DNA, might offer an advantage over exome sequencing if one is searching for short deletions spanning only a few hundred bp. It remains to be determined whether deletions affecting a single exon are more common than initially thought because they were missed by exome sequencing in some reports.

## Methods

### RNA isolation

Total RNA was isolated from the blood of the patients using the QIAamp RNA Blood Mini kit from Qiagen (Hilden, Germany). Written consent was obtained to use the samples for genetic testing and to publish the results in accordance with the guidelines of the Swiss Society of Medical Genetics (www.sgmg.ch).

The blood (2 ml) was collected in the presence of the anti-coagulant EDTA and immediately processed. Erythrocytes were lysed in hypotonic buffer. Leukocytes were collected by centrifugation, lysed with a QIAshredder and dissolved in RLT buffer. Total RNA was adsorbed to a silica-based membrane, washed and selectively eluted with TE buffer. The purified RNA was transcribed into first-strand cDNA using hexamer primers and Improm-II Reverse Transcriptase (Promega, Madison WI, USA).

### Isolation of genomic DNA

DNA was isolated from freshly drawn blood using the Wizard genomic DNA purification kit from Promega. Red blood cells were lysed in Cell Lysis Solution. White blood cells were collected by centrifugation and lysed in Nuclei Lysis Solution. RNA was digested with RNase and cellular proteins were removed by salt precipitation. Finally, the genomic DNA was concentrated by precipitation with isopropanol and washed with ethanol.

### PCR amplification and DNA sequencing

Fragments of interest were amplified from the cDNA by PCR through 35 cycles (20″ at 95 °C, 20″ at individual annealing temperature (Additional file 1: Table S1), 15″ at 72 °C) with KAPA high fidelity DNA polymerase (KAPA Biosystems, Boston, USA). The fragments were resolved on 1 % agarose gels. Single bands were recovered from the agarose gel with the GeneElute extraction kit (Sigma, St. Louis MO, USA). For long-range PCR, the cycle number was reduced to 33 and the extension time at 72 °C was increased to 2′ 40″. In this case, the amplified fragments were resolved on a 0.5 % agarose gel. DNA sequences were determined by Sanger cycle sequencing on an ABI 3730 platform.

### Sequence analysis

All sequences were analysed with the program package of MacVector Inc. For sequence alignments, the program ClustalW was used with the following parameters: Scoring matrix Gonnet, open gap penalty 10, extend gap penalty 0.1. Transmembrane sequences and PFAM protein domains were identified with the simple modular architecture research tool (Schultz et al. 1998).

## Additional file

10.1186/s40064-016-1695-6 Additional file containing supplementary information (Figure S1 and Table S1).

## References

[CR1] Abeysinghe SS, Chuzhanova N, Krawczak M, Ball EV, Cooper DN (2003). Translocation and gross deletion breakpoints in human inherited disease and cancer I: nucleotide composition and recombination-associated motifs. Hum Mutat.

[CR2] Breton C, Imberty A (1999). Structure/function studies of glycosyltransferases. Curr Opin Struct Biol.

[CR3] Chen JM, Cooper DN, Férec C, Kehrer-Sawatzki H, Patrinos GP (2010). Genomic rearrangements in inherited disease and cancer. Semin Cancer Biol.

[CR4] Cheung PK, McCormick C, Crawford BE, Esko JD, Tufaro F, Duncan G (2001). Etiological point mutations in the hereditary multiple exostoses gene EXT1: a functional analysis of heparan sulfate polymerase activity. Am J Hum Genet.

[CR5] Esko JD, Selleck SB (2002). Order out of chaos: assembly of ligand binding sites in heparan sulfate. Annu Rev Biochem.

[CR6] Farach-Carson MC, Hecht JT, Carson DD (2005). Heparan sulfate proteoglycans: key players in cartilage biology. Crit Rev Eukaryot Gene Expr.

[CR7] Hall CR, Cole WG, Haynes R, Hecht JT (2002). Reevaluation of a genetic model for the development of exostosis in hereditary multiple exostosis. Am J Med Genet.

[CR8] Hecht JT, Hayes E, Haynes R, Cole WG, Long RJ, Farach-Carson MC, Carson DD (2005). Differentiation-induced loss of heparan sulfate in human exostosis derived chondrocytes. Differentiation.

[CR9] Hilton MJ, Gutiérrez L, Martinez DA, Wells DE (2005). EXT1 regulates chondrocyte proliferation and differentiation during endochondral bone development. Bone.

[CR10] Huegel J, Sgariglia F, Enomoto-Iwamoto M, Koyama E, Dormans JP, Pacifici M (2013). Heparan sulfate in skeletal development, growth, and pathology: the case of hereditary multiple exostoses. Dev Dyn.

[CR11] Jennes I, Entius MM, Van Hul E, Parra A, Sangiorgi L, Wuyts W (2008). Mutation screening of EXT1 and EXT2 by denaturing high-performance liquid chromatography, direct sequencing analysis, fluorescence in situ hybridization, and a new multiplex ligation-dependent probe amplification probe set in patients with multiple osteochondromas. J Mol Diagn.

[CR12] Jennes I, Pedrini E, Zuntini M, Mordenti M, Balkassmi S, Asteggiano CG, Casey B, Bakker B, Sangiorgi L, Wuyts W (2009). Multiple osteochondromas: mutation update and description of the multiple osteochondromas mutation database (MOdb). Hum Mutat.

[CR13] Jennes I, de Jong D, Mees K, Hogendoorn PC, Szuhai K, Wuyts W (2011). Breakpoint characterization of large deletions in EXT1 or EXT2 in 10 multiple osteochondromas families. BMC Med Genet.

[CR14] Kobayashi S, Morimoto K, Shimizu T, Takahashi M, Kurosawa H, Shirasawa T (2000). Association of EXT1 and EXT2, hereditary multiple exostoses gene products, in Golgi apparatus. Biochem Biophys Res Commun.

[CR15] Krawczak M, Cooper DN (1991). Gene deletions causing human genetic disease: mechanisms of mutagenesis and the role of the local DNA sequence environment. Hum Genet.

[CR16] Lin X, Wei G, Shi Z, Dryer L, Esko JD, Wells DE, Matzuk MM (2000). Disruption of gastrulation and heparan sulfate biosynthesis in Ext1-deficient mice. Dev Biol.

[CR17] McCormick C, Duncan G, Goutsos KT, Tufaro F (2000). The putative tumor suppressors EXT1 and EXT2 form a stable complex that accumulates in the Golgi apparatus and catalyzes the synthesis of heparan sulfate. Proc Natl Acad Sci USA.

[CR18] Miller JN, Pearce DA (2014). Nonsense-mediated decay in genetic disease: friend or foe?. Mutat Res, Rev Mutat Res.

[CR19] Nadanaka S, Kitagawa H (2008). Heparan sulphate biosynthesis and disease. J Biochem.

[CR20] Pedersen LC, Dong J, Taniguchi F, Kitagawa H, Krahn JM, Pedersen LG, Sugahara K, Negishi M (2003). Crystal structure of an alpha 1,4-N-acetylhexosaminyltransferase (EXTL2), a member of the exostosin gene family involved in heparan sulfate biosynthesis. J Biol Chem.

[CR21] Pedrini E, Jennes I, Tremosini M, Milanesi A, Mordenti M, Parra A, Sgariglia F, Zuntini M, Campanacci L, Fabbri N, Pignotti E, Wuyts W, Sangiorgi L (2011). Genotype-phenotype correlation study in 529 patients with multiple hereditary exostoses: identification of “protective” and “risk” factors. J Bone Joint Surg Am.

[CR22] Schultz J, Milpetz F, Bork P, Ponting CP (1998). SMART, a simple modular architecture research tool: identification of signaling domains. Proc Natl Acad Sci USA.

[CR23] Senay C, Lind T, Muguruma K, Tone Y, Kitagawa H, Sugahara K, Lidholt K, Lindahl U, Kusche-Gullberg M (2000). The EXT1/EXT2 tumor suppressors: catalytic activities and role in heparan sulfate biosynthesis. EMBO Rep.

[CR24] Stickens D, Zak BM, Rougier N, Esko JD, Werb Z (2005). Mice deficient in Ext2 lack heparan sulfate and develop exostoses. Development.

[CR25] Szuhai K, Jennes I, de Jong D, Bovée JV, Wiweger M, Wuyts W, Hogendoorn PC (2011). Tiling resolution array-CGH shows that somatic mosaic deletion of the EXT gene is causative in EXT gene mutation negative multiple osteochondromas patients. Hum Mutat.

[CR26] Waaijer CJ, Winter MG, Reijnders CM, de Jong D, John Ham S, Bovée JV, Szuhai K (2013). Intronic deletion and duplication proximal of the EXT1 gene: a novel causative mechanism for multiple osteochondromas. Genes Chromosom Cancer.

[CR27] Zak BM, Schuksz M, Koyama E, Mundy C, Wells DE, Yamaguchi Y, Pacifici M, Esko JD (2011). Compound heterozygous loss of Ext1 and Ext2 is sufficient for formation of multiple exostoses in mouse ribs and long bones. Bone.

