# Supplementary material for: Deletion of exon 8 from the EXT1 gene causes multiple osteochondromas (MO) in a family with three affected members
Source: Springerplus. 2016 Jan 22;5:71. doi: 10.1186/s40064-016-1695-6 (PMC4723372; doi:10.1186/s40064-016-1695-6)
Supplement: Supplementary file 1 — 10.1186/s40064-016-1695-6 Additional file containing supplementary information (Figure S1 and Table S1). [file 40064_2016_1695_MOESM1_ESM.pdf]

Supplementary Information to:

Deletion of exon 8 from the EXT1 gene causes multiple osteochondromas (MO) in a family with three affected members

Lei Zhuang, Simon D. Gerber, Stefan Kuchen, Peter M. Villiger and Beat Trueb

Department of Clinical Research, University of Bern, 3008 Bern, Switzerland
E-mail: beat.trueb@dkf.unibe.ch

Fig. S1 Domain structure of the five members of the exostosin family. The protein sequences of human EXT1 (NP_000118), EXT2 (NP_997005), EXTL1 (NP_004446), EXTL2 (NP_001430) and EXTL3 (NP_001431) are depicted. Transmembrane sequences (grey boxes), Exostosin interaction domains (PF03016) and Glycosyltransferase 64 domains (PF09258) were identified by SMART. Numbers give beginning and end of the amino acid sequences. The figure is approximately drawn to scale

Figure S1

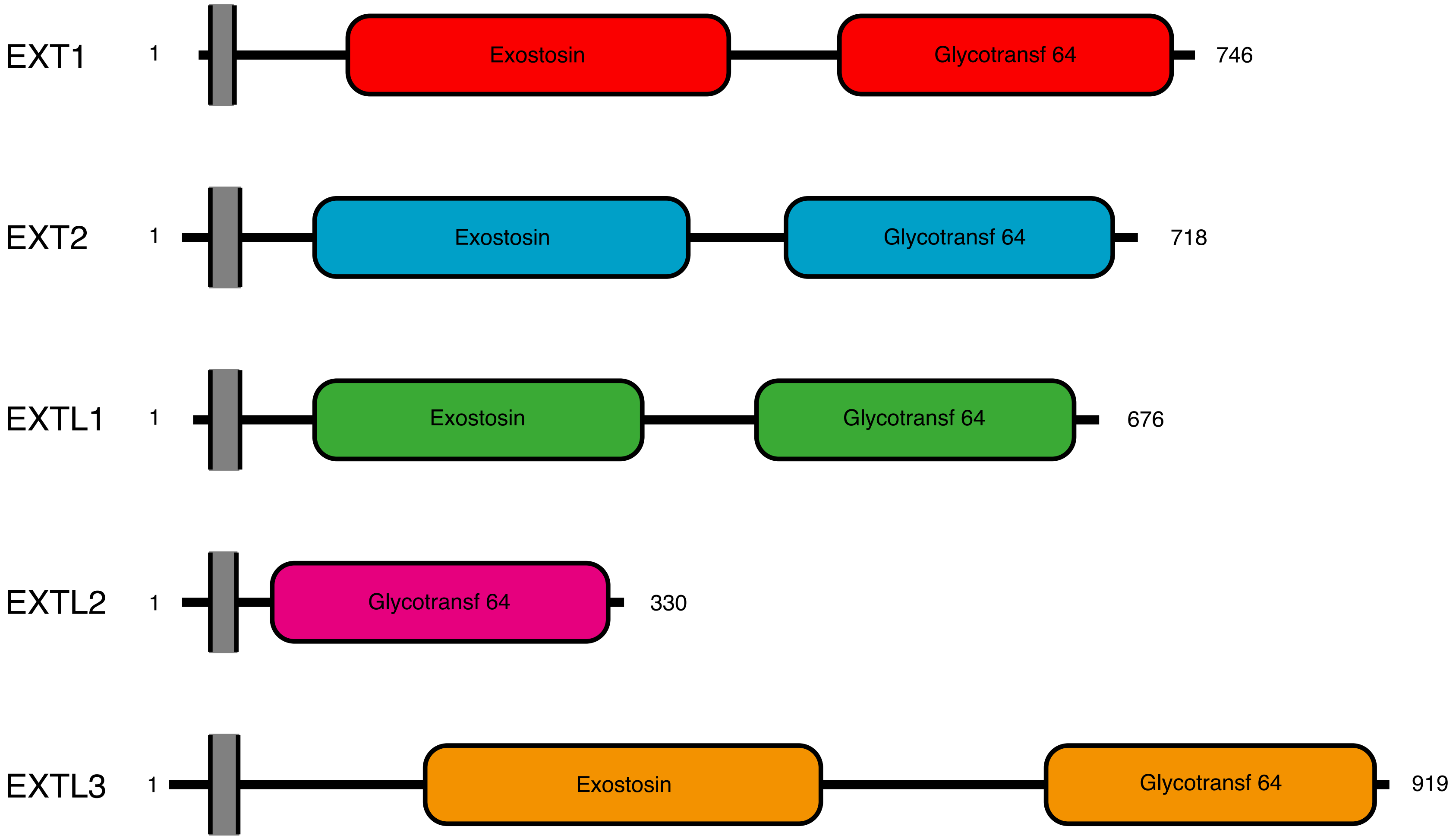
EXT1
1
Exostosin
Glycotransf 64
746
EXT2
1
Exostosin
Glycotransf 64
718
EXTL1
1
Exostosin
Glycotransf 64
676
EXTL2
1
Glycotransf 64
330
EXTL3
1
Exostosin
Glycotransf 64
919

Table S1 Primers used in this study

Name	Sequence 5' to 3'	Purpose	Product bp	5' Position	Annealing T
Ext1 Up2	ATTTACAATCCTCTTGACCCAG	EXT1 cDNA	382	0743	59
Ext1 Low21	GTAGACTTTGAAGCCGTTTTTC			1124	
Ext1 Up21	GAAAAACGGCTTCAAAGTCTAC	EXT1 cDNA	398	1103	58
Ext1 Low2	TGGGATGATCCTTAGAAAAGAG			1500	
Ext1 Up1	CCAAAGCCAGCATCAGTACTG	EXT1 cDNA	583	1423	60
Ext1 Low1	AAGAATTGTGTCTGCTGTCTAAGTG			2005	
Ext1 Up3	GATTAATTGGAACCAAGCTGCC	EXT1 cDNA	519	1886	60
Ext1 Low103	TTGCTCTCTCCTTCAATGACGAC			2404	
Ext1 Up103	AATTCTTGTGGGAGGCTTATTTTTC	EXT1 cDNA	406	1999	59
Ext1 Low103	TTGCTCTCTCCTTCAATGACGAC			2404	
Ext1 Up5	GCTGTGCCTGTCGTCGTCATTG	EXT1 cDNA	354	2370	62
Ext1 Low5	GGCCAATTGGTCCACCATGTTC			2723	
Ext1 Up4	TCCCATTACCTGCCAGCCAGC	EXT1 cDNA	357	2676	60
Ext1 Low4	CCCACTCAGCCGGATTCCTCA			3032	
Ext1 Up8	TGACCACTTTGCCCAGCGACAGAGCT	EXT1 cDNA	207	2858	62
Ext1 Low8	GCTTGACCCCCATCCCTTCTTG			3064	
Ext2 Up1	TGGCCGAGGAGTGTGAGGAAGA	EXT2 cDNA	789	0297	63
Ext2 Low1	GCCGTGGACCTGGTCCTTTCTC			1085	
Ext2 Up2	ATAGTCCACTGTCAGCTGAGGT	EXT2 cDNA	759	1032	59
Ext2 Low2	GTAGTTTGGATAGACTGGGCAC			1790	
Ext2 Up3	AGAGAGCCTCTTCCGGGTCATC	EXT2 cDNA	813	1732	60
Ext2 Low3	TGTTCTCTCCCTCTGTCCCAGC			2544	
Exon 8 Up2	CCTCTATGGTACATTCAACACAAAGGTTTAAG	Ex8 splice sites	2573	117814367	61
Exon 8 Low2	TAGTAGAGATAAGGTTTCACCATATTGGCCAG			117811795	
Intron789 Up1	TGTGGAACCACTGTGGAGCCTTG	EXT1 gene	8037	117818411	58
Intron789 Low3	GCAGGCAAAAGCAAACCAGACTG			117810375	
Intron789 Up7	CTTCCTCATTCTCCATTTTAAAATCTCTATCT	EXT1 gene	5633	117815339	61
Intron789 Low7	GAGCACGTTATCTCAAAGTTAAAAGGTAG			117809707	
